# *Helicobacter pylori* Infection in Dialysis Patients: A Meta-Analysis

**DOI:** 10.1155/2013/785892

**Published:** 2013-11-07

**Authors:** Min Gu, Shuping Xiao, Xiaolin Pan, Guoxin Zhang

**Affiliations:** ^1^Department of Gastroenterology, First Affiliated Hospital of Nanjing Medical University, Nanjing 210029, China; ^2^First Clinical Medical College of Nanjing Medical University, Nanjing 210029, China

## Abstract

*Background*. Infection with *Helicobacter pylori* contributes to the etiopathogenesis of various extragastrointestinal conditions, yet its etiological association with either symptomatic or asymptomatic dialysis patients remains inconclusive. *Methods*. Two researchers working independently conducted a literature search of the online databases PubMed, EMBase, ScienceDirect, and Cochrane Central Register of Controlled Trials to identify relevant articles to the end of 2012. Case-control and cross-sectional studies that met the inclusion criteria were included. *Results*. Fifteen studies involving 1237 dialysis patients and 1568 controls with normal renal function were included. Compared with normal controls, dialysis patients overall were associated with a relatively lower risk of *H. pylori* infection though not statistically significant. A significant inverse association was found between *H. pylori* prevalence and duration of treatments in those who were dialyzed >4 years (odds ratio 0.28; 95% confidence interval 0.22–0.36, *P* < 0.00001). No relationship between *H. pylori* status and duration of dialysis was observed in CRF patients. There were no significant differences in endoscopic features between patients and controls. *Conclusions*. Our meta-analysis found no evidence of a significant association between infection with *H. pylori* and dialysis overall, whereas long-term treatments of more than four years had a significant protective effect.

## 1. Introduction

*Helicobacter pylori*, an infectious organism, is present in about 50% of the global population, and the infection levels exceed 70% in some developing areas [1]. Infection with *H. pylori* has been implicated not only in the etiopathogenesis of gastrointestinal disease, such as gastritis, ulcerative diseases, low-grade mucosa-associated lymphoid tissue lymphoma, and gastric malignancies [2], but also in various extragastrointestinal conditions, among them chronic renal disease [3]. 

From 25% to 75% of chronic renal failure (CRF) patients who receive hemodialysis or continuous ambulatory peritoneal dialysis (CAPD) for long periods experience gastrointestinal troubles [4]. It has been postulated that high urea concentration makes the gastric mucosa of these patients more susceptible to colonization by *H. pylori* [5]. However, an etiological association between *H. pylori* and either symptomatic or asymptomatic dialysis patients remains inconclusive. 

The prevalence of *H. pylori* infection in CRF patients may be as high as 64% and significantly higher in dialysis patients than in normal controls [6–9]. Others [10–12] report quite the opposite. Many factors would seem to contribute to the inhibition of *H. pylori* growth in the stomach of CRF patients (e.g., higher levels of proinflammatory cytokines, impaired immune system, increased pH, higher blood urea levels, and antibiotic treatment). Nevertheless, some studies [13–19] found no difference in the prevalence of *H. pylori* infection between patients on dialysis and healthy controls, leading to the conclusion that the level of urea is not a risk factor predisposing to *H. pylori* infection in this population. Because of these conflicting reports, the seriousness of *H. pylori* infection in dialysis patients remains unclear. 

The number of dialysis patients increases by 7% annually [20], and it is therefore imperative to resolve some important issues concerning *H. pylori *infection in dialysis patients. The present study is a meta-analysis, designed to help clarify the prevalence of* H. pylori *in CRF patients as well as the relationship between dialysis duration and the prevalence of *H. pylori*. In addition, *H. pylori* status in CRF patients and the course of dialysis will be discussed.

## 2. Materials and Methods

### 2.1. Literature Sources and Searches

We systematically searched the databases MEDLINE, EMBASE, ScienceDirect, and Cochrane Central Register of Controlled Trials (CENTRAL) for relevant articles and abstracts published in English (ending 31 December 2012). Terms and keywords used to identify articles in Medical Subject Headings (MeSH) were *Helicobacter pylori*/*H. pylori*, and dialysis ((“*Helicobacter pylori*” OR “*H. pylori*”) AND “dialysis”). Two reviewers (MG and SPX) manually screened each eligible article's title, abstract, and full text to independently determine if the article met the inclusion criteria (below). Differences between the reviewers were solved by consensus.

### 2.2. Inclusion and Exclusion Criteria

For inclusion in the meta-analysis, case-control or cross-sectional studies had to report data on the rate of *H. pylori* infection in patients with and without dialysis and include a control group with normal renal function; base diagnosis of *H. pylori* infection on histology (e.g., Giemsa stain or Warthin-Starry method), culture, immunoglobulin G (IgG) antibody detection, rapid urease test, or urea breath test; concern human subjects only; and be published in English. Studies were excluded that were case reports, observational studies without control groups, review of the literature, or duplicated reports; if data on *H. pylori* infection in the dialysis group or control group was incomplete or unavailable; or if subjects had a history of drug use for antibiotics, H2 blockers, proton pump inhibitors, or bismuth within 4 weeks.

### 2.3. Data Extraction

Two independent reviewers extracted the information from the included articles. Discrepancies in the extraction were resolved by mutual discussion. For each study, the following data were collected: author; publication year; country; study design; basic characteristics of patients including number of patients with and without dialysis and type and duration of dialysis; detection methods for *H. pylori* infection and endoscopic abnormalities.

### 2.4. Data Analysis

The software Review Manager (RevMan, version 5.1, Copenhagen: The Nordic Cochrane Centre, The Cochrane Collaboration, 2011) was used to analyze the data. We arranged eligible articles chronologically, starting with the earliest. The odds ratios (ORs) and their 95% confidence intervals (CIs) for major outcomes were estimated in a fixed model or random model for each study. Statistical heterogeneity was evaluated with the *I* ^2^ statistic, and *I* ^2^ > 50% indicated substantial heterogeneity [29], in which case the condition random effects model was used. The differences were considered statistically significant when a *P* value was less than 0.05. 

## 3. Results

### 3.1. Basic Information and Characteristics

**Figure 1:**
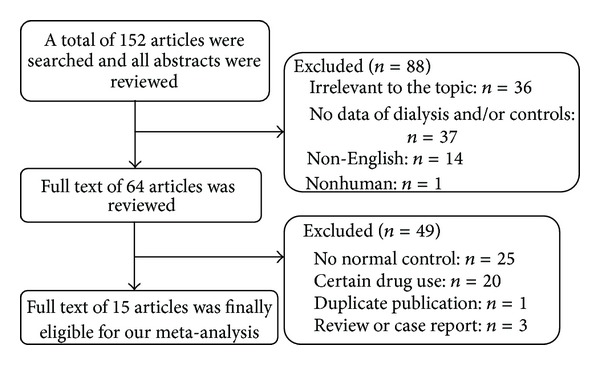
Flow chart of the eligibility selection process.

The literature search initially yielded 152 articles relevant to the topic (Figure 1). Eighty-eight of these were excluded for not meeting the inclusion criteria. The full texts of the remaining 64 citations were carefully reviewed. Ultimately, 49 of the 64 were excluded due to the use of certain drugs within 4 weeks or for meeting any other of the exclusion criteria. This process left 15 qualified essays (Table 1).

### 3.2. Overall Analysis

**Figure 2:**
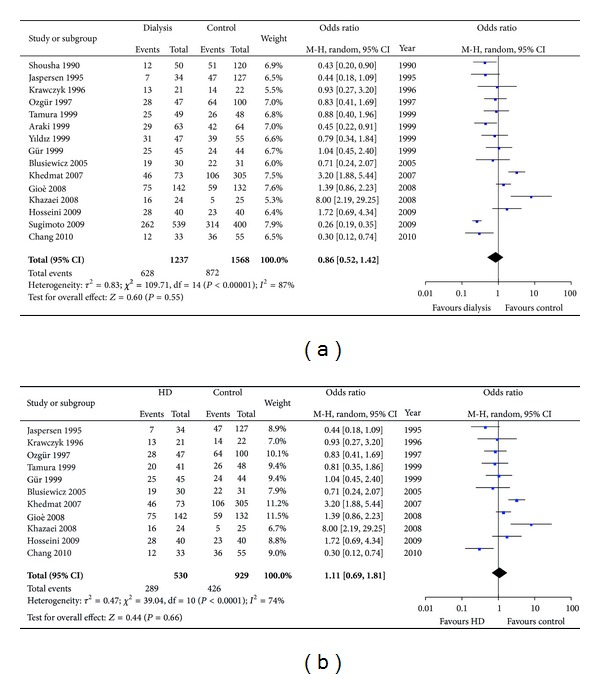
(a) Prevalence of *H. pylori* in dialysis patients and controls with normal renal function. (b) The prevalence of *H. pylori* in hemodialysis patients and controls with normal renal function.

**Figure 3:**
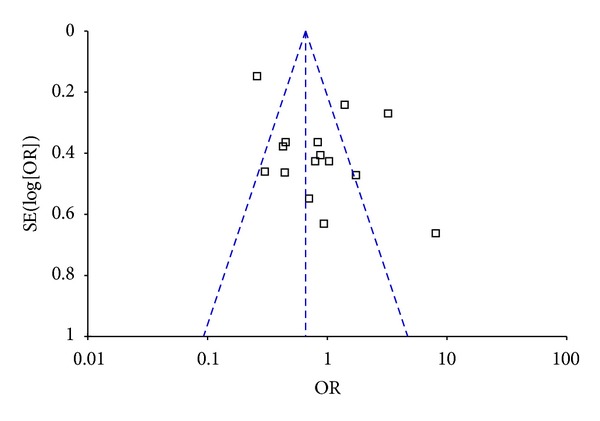
Funnel plot for 15 studies.

These 15 articles comprised 1237 dialysis patients and 1568 controls with normal renal function. Since *I* ^2^ was greater than 50%, a random model was applied. Pooled data showed that there was no difference in *H. pylori* prevalence between the dialysis (hemodialysis and CAPD) patients and normal controls (OR = 0.86, 95% CI: 0.52–1.42, *P* = 0.55; Figure 2(a)). A subanalysis showed no difference in *H. pylori* infection between patients receiving hemodialysis and the control group (OR = 1.11, 95% CI: 0.69–1.81, *P* = 0.66; Figure 2(b)). A funnel plot indicated that there was no publication bias (Figure 3).

**Figure 4:**
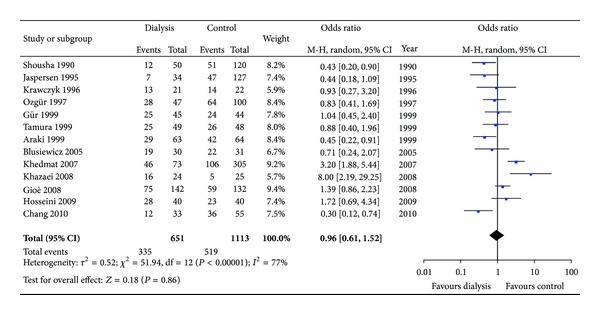
Various methods for detecting *H. pylori* infection (excluding IgG titer).

In this meta-analysis, various methods were adopted to confirm *H. pylori* infection as stated previously. As we all know, IgG antibody detection cannot judge present infection of *H. pylori*, since serum antibodies specific to *H. pylori* would still remain for several months after successful eradication, nevertheless; serology is the only test which is not affected by local changes in the stomach that could lead to a low bacterial load and to false negative results of the other tests and it is the third method commonly used as a noninvasive method to diagnose* H. pylori *infection [30]. In order to exclude the probability that different methods for *H. pylori* detection would lead to different outcomes, we chose to exclude 2 articles [11, 22] which only utilized IgG to detect *H. pylori* infection. However, subsequent analysis again found no differences between the two groups (Figure 4). Still, we wanted to detect if other detection methods like rapid urease test (RUT) would influence the overall analysis, while more than one kind of detection method was involved in the other studies included in our meta-analysis. The data of other detection methods cannot be analyzed separately.

### 3.3. Effect of Dialysis Duration on *H. pylori* Prevalence

**Figure 5:**
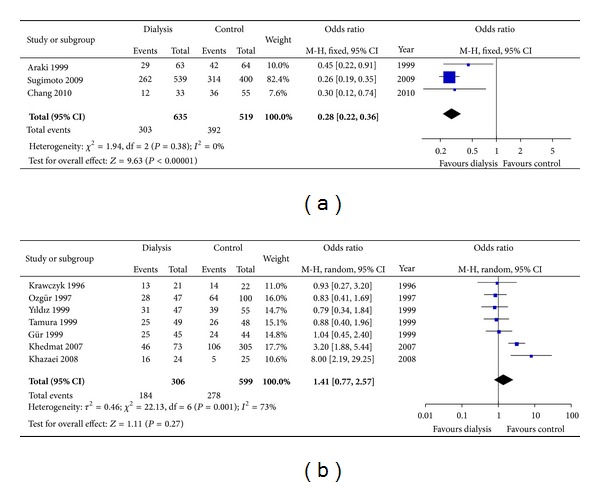
(a) Effect of dialysis duration (>4 years) on *H. pylori *prevalence. (b) Effect of dialysis duration (≤4 years) on *H. pylori* prevalence.

Some studies have indicated that the rate of *H. pylori* infection decreases over a prolonged course of hemodialysis. Hence we performed a subgroup meta-analysis of *H. pylori* infection and the duration of dialysis (Figures 5(a) and 5(b)). Those who underwent dialysis longer than four years [4, 11, 28] indeed showed a significantly lower rate of *H. pylori* infection (*P* < 0.00001) than those with normal renal function, while it is another story when it comes to those who endured dialysis duration shorter than four years [6, 14, 15, 21–23, 25] (*P* = 0.27) with no difference in *H. pylori *infection rate between two groups.

### 3.4. Effect of *H. pylori* Status on Duration of Dialysis

**Figure 6:**
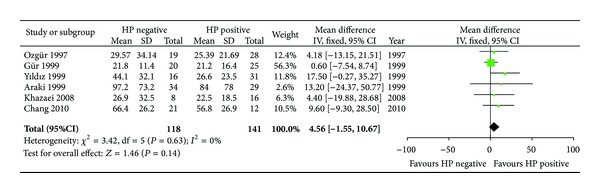
Association between *H. pylori* status and duration of dialysis in CRF patients.

A few previous studies have shown that *H. pylori* positive patients required a significantly shorter course of dialysis than uninfected patients [22, 31]. Among the included studies, five studies [4, 15, 21, 22, 25, 28] evaluated the relationship between *H. pylori *status and duration of dialysis. However, no statistical significance was observed between *H. pylori* negative and* H. pylori* positive patients. The weighted mean difference between these studies was 4.56 (95% CI: −1.55–10.67, *P* = 0.14) (Figure 6). 

### 3.5. Endoscopic Findings

**Figure 7:**
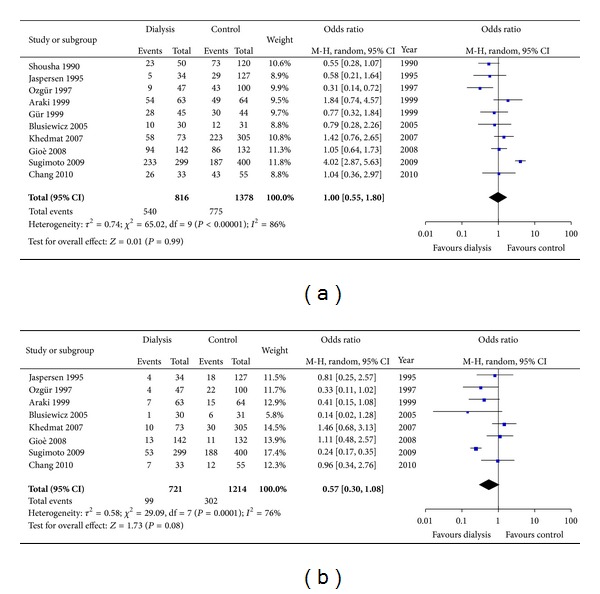
(a) Incidence of gastritis between CRF patients on dialysis and normal controls. (b) Incidence of ulcer diseases between CRF patients on dialysis and normal controls.

We compared the endoscopic findings between the dialysis and control groups, mainly concerning gastritis and ulcerative diseases. Ten studies [4, 6, 10, 11, 13, 15, 21, 24, 26, 28] provided detailed endoscopic information regarding, for example, gastritis, ulcerative diseases, and intestinal metaplasia. The incidence of gastritis and ulcerative diseases in dialysis patients and normal controls was 66.2% versus 56.2% (*P* = 0.99) and 13.7% versus 24.9% (*P* = 0.08), respectively. There are no significant differences in endoscopic abnormities between the dialysis patients and the controls with normal renal function (Figures 7(a) and 7(b)). 

## 4. Discussion

Recently, more and more evidence has shown that *H. pylori* is related to extragastrointestinal diseases such as iron deficiency anemia, idiopathic thrombocytopenic purpura [32], and diabetes mellitus [33]. Moreover, patients with CRF usually suffer from systemic or local chronic circulatory failure (or both) [34], hypergastrinemia [21], high ammonia levels [35], and enhanced inflammation that facilitates *H. pylori* infection. In the present study, we performed a meta-analysis and found that CRF patients on dialysis treatment had an overall *H. pylori* infection rate of ~50.8%, which was relatively but not significantly lower than the 55.6% in controls (*P* = 0.55). 

Upon investigating the association between *H. pylori* infection and the different types of dialysis, we found that *H. pylori* infection was not statistically associated with hemodialysis specifically. However, the *H. pylori* infection rate in the hemodialysis group (54.5%) was slightly higher than that of the control (45.9%), which contrasts with the results of the overall analysis. Due to lack of data, we were not able to analyze the difference in *H. pylori* prevalence between CRF patients undergoing CAPD and those receiving hemodialysis. Thus, our results from these studies revealed that the prevalence of *H. pylori* infection is similar between CRF patients who were receiving dialysis and the control group with normal renal function (*P* > 0.05). 

From the results of the present study, it appears that CRF treatment with dialysis does not change the probability of *H. pylori* infection. Although one researcher went against current thought and concluded that the level of urea is not a risk factor in *H. pylori* colonization [28], neither theory has proved definitive and more research is required to clarify the issue. Among the included studies in our meta-analysis, some researchers [4, 11, 13, 28] found that the prevalence of *H. pylori* in CRF patients undergoing dialysis was significantly lower than in non-CRF controls with or without gastrointestinal symptoms. The truth is that many CRF patients who receive dialysis inevitably have access to antibiotics, proton pump inhibitors, or H2 receptor antagonists which then influence the *H. pylori* infection rate to some extent [13, 36]. Moreover, gastric atrophy progresses along with decreased secretion of acid [37] as well as higher levels of proinflammatory cytokines [38] in CRF patients, making *H. pylori* difficult to survive. Apart from these, the prevalence of *H. pylori* varies widely across different demographic and geographic areas due to economic situations, sanitary conditions, cultural habits, and more. 

Our subgroup analysis revealed that the prevalence of *H. pylori* of those patients who were on dialysis for longer than 4 years was significantly lower than of individuals with normal renal function, while the duration of dialysis between *H. pylori* negative and *H. pylori* positive patients did not differ from each other. It is in accordance with other studies. Sugimoto et al. [11] showed that the prevalence of *H. pylori* infection decreased in the first 4 years of dialysis and plateaued after 5 years of treatment and it was not affected by basement diseases. He and his colleagues concluded that more than one-third of patients who had received approximately four years of dialysis treatments had been naturally cured of *H. pylori* infections. Nakajima et al. [31] also reported that the prevalence of *H. pylori* decreased along with extended hemodialysis duration of two years and more. They declared that the reduction of *H. pylori* prevalence in long-term dialysis patients was due to reduction of gastric acid secretion related to chronic gastritis or frequent antibiotic consumption. Nevertheless there are actually conflicts about the relationship between *H. pylori* status and duration of dialysis. Several studies argued that duration of dialysis was inversely related to *H. pylori* colonization in dialysis patients [39–41], and some found an opposite result [42]. Yet, the underlying mechanism is still obscure. More investigations are warranted to be conducted to elucidate these findings in the future. 

Endoscopic abnormities such as erosive gastritis, duodenitis, and peptic ulcers are often found in CRF and dialysis patients. In some studies, peptic ulcers and gastroduodenal mucosa lesions were associated with *H. pylori* infection [43–47]. Khedmat et al. [6] showed that there was no significant difference in the rate of nonerosive gastritis, duodenitis, and gastric ulcer diseases between hemodialysis patients and those with normal renal function. These findings are in accordance with the results of our present meta-analysis, which indicated no statistical differences between dialysis patients and normal controls (*P* > 0.05) concerning endoscopic gastritis and ulcerative diseases. Thus it seems that dialysis itself is not a risk factor for the occurrence of gastritis or ulcers, although it is still necessary to perform endoscopy in dialysis patients with gastrointestinal symptoms. However, the above results rarely came from children's studies. Whether to recommend upper gastrointestinal examination based on symptoms requires more consideration in pediatric dialysis patients.

Although in the present meta-analysis we found no significant difference in *H. pylori *prevalence between dialysis patients and control subjects, according to some studies [21, 48, 49] successful eradication of *H. pylori* would lead to a significant decrease in dyspeptic symptoms, improvement in upper endoscopic results, and reduction in serum gastrin concentrations among hemodialysis patients. In such patients, due to impaired renal function and decline in the rate of excretion of drugs, the ideal treatment regimen should emphasize high efficacy and few adverse effects. Seyyedmajidi et al. conducted a randomized controlled trial comparing sequential therapy and standard triple therapy for *H. pylori* eradication in uraemic and nonuraemic patients. The eradication rates did not differ with both sequential and standard therapeutic regimens in the patients and normal controls. They preferred the standard triple therapy due to its lower side effects and complexity [50]. Chang et al. [28] found that a 7-day triple therapy with a low-dose OAC (omeprazole, amoxicillin, and clarithromycin) regimen was effective and safe for eradication of *H. pylori* infection in hemodialysis patients, with the consideration that amoxicillin and clarithromycin are primarily eliminated via the renal route. Further studies investigating the effect of eradication of *H. pylori *on symptom relief of dialysis patients are necessary.

When weighing the findings of the present meta-analysis, it is imperative to note that these studies were all case-control or cross-sectional studies, each performed at a single center with a cohort, and the sociodemographic characteristics of the populations were unclear. Although we adjusted for potential confounders, heterogeneity still existed among the study designs; confounding is an intrinsic limitation of these observational studies, so we precluded any assessment of causality in reported associations. Also, variables such as age and gender may be important considerations in the analysis of risk factors, but here we were unable to adjust for them, mainly due to incomplete data. 

## 5. Conclusion

In the present meta-analysis there was no evidence of a significant association between infection with *H. pylori* and dialysis treatments for CRF patients. With heterogeneity limiting certainty of this association, there is a need for well-conducted randomized controlled trials to further verify these findings. According to subgroup analysis dialysis treatments for more than 4 year appears to have a protective effect against *H. pylori* infection; mechanistic studies of this negative association are needed to be further identified. It is indeterminable whether *H. pylori* status would affect duration of dialysis in CRF patients or whether endoscopic abnormalities of dialysis patients are related to *H. pylori* infection; further clinical studies investigating the effect of *H. pylori *infection on endoscopic findings of dialysis patients are necessary.

## Figures and Tables

**Table 1:** Basic information of eligible articles.

Author (ref.)	Year	Country	Study design	Age, y	Test confirming infection	Duration of dialysis, m	Dialysis type, *n*		HP(+), *n*	
							HD	CAPD	Controls	Dialysis
Shousha et al. [13]	1990	UK	Case-control	HP(+) 54 ± 14.3 HP(−) 48 ± 14.2	Warthin-Starry, Giemsa	NG	NG	NG	51/120	12/50
Jaspersen et al. [10]	1995	Germany	Case-control	58.2 ± 12.6	Urease test, Giemsa	NG	7/34	0	47/127	7/34
Krawczyk et al. [14]	1996	Poland	Case-control	36.8 ± 13.2	Urease test, Giemsa	28 ± 12.2	13/21	0	14/22	13/21
Ozgür et al. [15]	1997	Turkey	Case-control	37.27 ± 14.08	Urease test	28.87 ± 28.92	28/47	0	64/100	28/47
Gür et al. [21]	1999	Turkey	Case-control	HP(+) 35.1 ± 4.2 HP(−) 32.5 ± 5.3	Urease test, histology	HP(+) 21.2 ± 16.4 HP(−) 21.8 ± 11.4	25/45	0	24/44	25/45
Araki et al. [4]	1999	Japan	Case-control	57.4 ± 12.8	Histology, culture	91.2 ± 62.4	NG/54	NG/9	42/64	29/63
Yildiz et al. [22]	1999	Turkey	Cross-sectional	36.6 ± 15.2	ELISA (IgG)	32.5 ± 27.7	NG	NG	39/55	31/47
Tamura et al. [23]	1999	Japan	Case-control	52.2 ± 1.8	Urease test, histology, and culture	29.3 ± 5.4	20/41	5/8	26/48	25/49
Blusiewicz et al. [24]	2005	Poland	Case-control	50.8 ± 2.9	Urease test, histology	NG	19/30	0	22/31	19/30
Khedmat et al. [6]	2007	Iran	Case-control	47.9 ± 3.5	Urease test	46.9 ± 10.7	46/73	0	106/305	46/73
Khazaei et al. [25]	2008	Iran	Case-control	14.7 ± 3.4	Urease test, histology	HP(+) 22.5 ± 18.5 HP(−) 26.9 ± 32.5	16/24	0	5/25	16/24
Gioè et al. [26]	2008	Italy	Case-control	NG	RUT, Giemsa	NG	75/142	0	59/132	75/142
Asl and Nasri [27]	2009	Iran	Cross-sectional	56 ± 14	Giemsa	≥6	28/40	0	23/40	28/40
Sugimoto et al. [11]	2009	Japan	Case-control	58.8 ± 0.4	ELISA (IgG)	100.8 ± 3.6	NG	NG	314/400	262/539
Chang et al. [28]	2010	Korea	Case-control	62 ± 9.8	RUT, histology	HP(+) 56.8 ± 26.9 HP(−) 66.4 ± 26.1	12/33	0	36/55	12/33

Ref.: reference; HD: hemodialysis; CAPD: continuous ambulatory peritoneal dialysis; NG: not given; RUT: rapid urease test.
